# Road toward institutionalizing health technology assessment in the Emirate of Abu Dhabi: The role of evidence-informed deliberative processes

**DOI:** 10.1017/S0266462324004744

**Published:** 2024-12-18

**Authors:** Leon Bijlmakers, Pinar Egeli, Amna Ibrahim Al Saeedi, Bakr Sadoon, Dirk Richter, Wija Oortwijn

**Affiliations:** 1 Radboud University Medical Centre, Nijmegen, The Netherlands; 2 Abu Dhabi Department of Health, United Arab Emirates

**Keywords:** health technology assessment, Abu Dhabi, evidence-informed deliberative processes, institutionalization

## Abstract

**Objective:**

This paper reports on the process used to embark on one of the core strategies of Abu Dhabi’s Department of Health, which was to develop a roadmap for HTA implementation and institutionalization, based on the aspirations and needs of local stakeholders and making use of the evidence-informed deliberative processes framework. The paper also highlights the main features of the road map that may be expected to address some of the current challenges.

**Methods:**

A series of activities were undertaken that informed the subsequent development of the roadmap. They comprised a situation analysis using a combination of desk research and semistructured (group) interviews with 45 stakeholders. The findings were discussed in two workshops; face-to-face with nonindustry stakeholders from Abu Dhabi, and online with industry representatives.

**Results:**

Guided by the EDP framework, the roadmap provides instructions how to organize stakeholder involvement, how to identify and operationalize decision criteria, and how to ensure that the decision-making process is transparent. Specific guidance is given on establishing an HTA structure with an appropriate policy framework, the formulation of an HTA program, a communication strategy, as well as building and leveraging HTA expertise.

**Conclusion:**

Broad stakeholder consultation has been instrumental toward the establishment of a comprehensive HTA framework in Abu Dhabi, and the development of a road map. The interest raised during stakeholder consultations and the commitments made hold promise for the adoption and establishment of EDP principles to support HTA in Abu Dhabi that have potential to contribute to a sustainable high-quality healthcare system.

## Introduction

Health technology assessment (HTA) is a multidisciplinary process that countries can use to navigate complex health-related policy making, set national priorities, and allocate scarce resources (1). Worldwide, the role of HTA is emerging fast, including in the Middle East and North Africa (2;3). In Jordan, for example, HTA is being institutionalized, through HTA capacity building and guideline development, amongst others, resulting in increased use of HTA for pricing and reimbursement decisions (4–6). Egypt has established a dedicated authority for Unified Procurement, Medical Supply, and Management of Medical Technology (7). Its role is to evaluate health technologies and public health programs, with a particular focus on the assessment of new pharmaceuticals introduced to the Egyptian market. The National Authority for Assessment and Accreditation in Healthcare (INEAS) in Tunisia is a long-standing member of the International Network of Agencies for Health Technology Assessment (INAHTA). INEAS actively promotes HTA, also in other countries in the region, through knowledge transfer, dissemination, and partnerships, with recently a special focus on promising innovative technologies that are costly and likely to have a major budget impact (8;9).

In the United Arab Emirates (UAE), of which Abu Dhabi forms part, the establishment of HTA is high on the agenda of a variety of stakeholders as recently demonstrated by Ahmad et al. (10). This relates to the prevailing challenges in sustainable health resource management in a context of high and fast rising healthcare expenditure due to a combination of demographic and epidemiological transition and medical tourism. The same authors argue, however, that “adopting HTA principles to lead the decision-making process in the UAE’s health system is still in a nascent stage.” In line with several others, including experts from the World Health Organization (WHO) (11), they further state that for HTA institutionalization, a “crucial and basic step is to design a compelling HTA policy awareness program and ensure effective communication” (10).

In 2022, the Department of Health (DoH) in Abu Dhabi, the capital and largest of the seven Emirates that constitute the UAE, set out to explore the development and adoption of an evidence-informed deliberative processes (EDPs) framework that would guide reimbursement and possible disinvestment decisions, using HTA. The EDP framework, described in a practical guide developed by Oortwijn et al. (12–14), may be used by both established and nascent HTA agencies to inform legitimate reimbursement decisions and guide wider health benefit package decisions. It consists of six steps: (A) Installing an advisory committee for health benefit package decisions; (B) defining decision criteria for such decisions; (C) selecting health technologies (interventions) for assessment; (D) scoping, assessment, and appraisal of the selected health technologies; (E) communication of reimbursement or inclusion decisions/recommendations and mechanisms for appeal; and (F) monitoring and evaluation.

EDPs for health benefit package decisions are recommended by the WHO and other international agencies and have been implemented successfully in a range of low-, middle-, and high-income countries (12;15;16). The underlying premise of EDPs is that stakeholder participation and transparency contribute to legitimizing the institution of HTA agencies, the HTA process, the recommendations, and the eventual decisions (i.e., (conditional) reimbursement or not, inclusion or exclusion of interventions regarding the basic health benefit package). Achieving this is often challenging for multiple reasons: (i) the complexity of the decisions that are required, for which a variety of factors and objectives need to be balanced, preferably through the involvement of a wide range of interest groups (stakeholders); (ii) the importance of legitimate decisions, as they may have a significant impact on population health and typically involve public financing; and (iii) the ethical and social considerations behind healthcare provision, which is widely perceived as a government duty, given that health is considered a public good or even a human right (17). This requires decision makers to be explicit and transparent about their decisions, which need to be based on actual needs and societal values, and to ensure consistency in how they arrive at these decisions (18).

The country-level operationalization of the EDP framework requires customization by taking into account the local context when implementing it in a stepwise manner. The success of applying EDPs, it is argued, strongly depends on the institutional structure of HTA, political will, and the HTA capacity that is locally available. This paper has a dual aim: firstly, it aims at describing the process used to codevelop a roadmap for strengthening Abu Dhabi’s institutional HTA structure; and secondly, it aims at highlighting the main features of the roadmap that is expected to support HTA implementation in line with the EDP framework for legitimate health benefit package decisions.

## Methods

The main activities to develop the roadmap included desk research, semistructured interviews with stakeholder groups, including policy makers, academia, service providers (health professionals), payers (insurance agencies), purchasers and industry representatives, and a national workshop to discuss the findings of the desk research and interviews.

The desk research, conducted in April–May 2022, comprised a review of health policy documents, available statistical data, and websites of the DoH and other government departments related to the organization of the health system and service provision in Abu Dhabi. This fed into a situation analysis of the policy landscape, sketching opportunities for strengthening HTA as an instrument to inform decision-making around the provision of health services and their coverage under health insurance. The situation analysis formed the basis for a series of seven group interviews and several individual interviews to take stock of stakeholder perspectives and interests and to assess the feasibility of an EDP approach to strengthening and institutionalizing HTA. The DoH identified and officially invited relevant stakeholders for the group interviews that were held at the DoH premises in Abu Dhabi during 1–2 June 2022. Stakeholders were categorized according to the seven P’s (policymakers, payers, product makers, principal investigators, patients and the public, providers, and purchasers) to ensure a balanced sample of stakeholder groups (19). Each group, except for product makers (industry), comprised between three and eight representatives. For product makers, a separate on-line group interview was held in September 2022 with nine members of the healthcare financing working group of Pharmaceutical Research and Manufacturers Association Gulf, which represents the leading innovative biopharmaceutical research companies in the Gulf region (20). The topic list for the semi-structured interviews, based on the EDP framework, is available as Supplementary file A. In total, 45 persons took part in the group interviews.

The combined results of the situation analysis and the first group interviews were presented and discussed in a half-day stakeholder workshop (June 3, 2022), which then informed the main building blocks of a draft roadmap. The building blocks are related to the six steps of the EDP framework and comprise the establishment of an HTA structure with an appropriate policy framework, formulation of an HTA program, communication, building, and leveraging HTA expertise.

The draft roadmap was discussed in a series of online meetings with senior DoH staff, agreed upon in October 2022, and formally approved by the DoH for implementation.

## Results

### Situation analysis

The latest available figures show that Abu Dhabi’s estimated population around mid-2016 was slightly more than 2.9 million (of which 63.9 percent were males), of which 19 percent were UAE nationals and 81 percent expatriates, with an average annual population growth rate of 5.6 percent for the years 2010–2016 (21). Ischemic heart disease, stroke, and kidney diseases form the top three causes of death in UAE as a whole, with high rates of lifestyle-related chronic conditions (22). Continuous demographic and epidemiological transitions and an accelerated introduction of new drugs and novel health technologies tend to increase healthcare expenditure (23;24).

In keeping with UAE’s Vision 2021 (25;26) and the Abu Dhabi Economic Vision 2030 (27), Abu Dhabi developed its own emirate-level vision, mission, and healthcare strategic plan (28). Based on five core values – Commitment to society, Creativity and innovation, Accountability, Integrity and Excellence – a set of strategic priorities were articulated, including Integrated continuum of care for individuals by the provision of the full spectrum of health services (priority #1), quality, safety and enhancing patient experience (#2), wellness and prevention – public health approach (#5), and sustainability of healthcare financial systems and ensuring value for money (#6) (28). HTA can be instrumental to help achieve these priorities.

The Emirate of Abu Dhabi has compulsory health insurance, with separate schemes and benefit packages for different target groups, including Thiqa, the government-funded, single-payer health insurance scheme, administered by the National Health Insurance Company Daman (Box 1). Of all government insurance contracts at the end of 2022, 27 percent covered national Thiqa members, 32 percent basic health insurance members, and 41 percent enhanced insurance members residing in Abu Dhabi. Around two-thirds of the latter market (Enhanced insurance) was held by three payers: Daman (49 percent), National Life (11 percent), and Oman Insurance (6 percent), with the remaining 34 percent shared by other payers that each had three percent or less market share.

Box 1: Health insurance schemes and benefit packages in the Emirate of Abu Dhabi*.

Thiqa, the government-funded, single-payer health insurance scheme is regulated by the DoH and administered by Daman, the National Health Insurance Company. Thiqa has different schedules of benefits for the following member categories:

- C1: UAE nationals and those of similar status to Abu Dhabi Emirate (broadest coverage),
- C2: UAE nationals and those of similar status of Northern Emirates ‘working and residing in owned properties’ in Abu Dhabi Emirate,
- C3: UAE nationals and those of similar status of Northern Emirates not residing and working in Abu Dhabi Emirate, and
- C4: Non-national members sponsored by UAE nationals’ families.

Non-nationals have two options to take out private health insurance:

- Basic health insurance for individuals with limited income in accordance with the threshold set by the Regulations (Health Insurance law 23/2005) and dependents of non-nationals who are ineligible for employment-based health insurance and
- Enhanced health insurance for individuals above the income threshold set by the Regulations for the basic health insurance (extended package for which a higher premium applies than for basic health insurance).

*Adapted from https://www.thiqa.ae/en/about-thiqa-programme/thiqa-programme-network

Similar to the increased healthcare spending globally, the Abu Dhabi government-funded benefit packages (Thiqa) are under continuous pressure to expand. Stakeholder interviews did indicate a general awareness that Abu Dhabi’s healthcare resources are finite and that it is essential to inform resource allocation decisions through evidence-informed mechanisms such as HTA. The stakeholder interviews and workshop revealed a broad consensus on the need to accelerate the use of HTA and that strengthening the existing HTA capacity is required to ensure equitable access to health technologies.

### Challenges in the HTA structure and processes already in place

The Abu Dhabi DoH does have regulation in place and a mechanism to grant market approval for new health technologies following applications submitted by manufacturers, UAE authorized distributors, or healthcare providers. The Research and Innovation Center performs this task. Once a new technology receives market approval, the Healthcare Payers Sector of the DoH prepares pricing and reimbursement recommendations for higher level approval.

The situation analysis and stakeholder consultations indicated the need for better coordination of HTA procedures and adoption of an evidence-informed framework to guide systematic pricing and reimbursement decisions. Since its establishment in 2019, the Centre of Research and Innovation in the DoH of Abu Dhabi had received approximately 110 applications for market approval of new pharmaceutical products over a period of 3 years; about 40 of these were approved, only six were disallowed. The remaining applications were still being processed (in June 2022), partly because of incomplete evidence, with increasing pressure to complete the approval process within 1 month after receipt of the application and proceeding to a pricing and reimbursement decision. While the large majority of applications (an estimated 90 percent; personal communication) had already been approved by the Food and Drug Administration in the United States and/or the European Medicines Agency, local approval was judged necessary mainly to reconfirm safety and clinical efficacy of the technology, and also to assess its added value compared to already existing products. Cost-effectiveness analysis was a relatively new task for the Healthcare Payers Sector, as this was previously done by Daman, the national health insurance agency. DoH staff responsible for HTA identified two main challenges. Firstly, insufficient capacity both in terms of number of available staff and their qualifications to evaluate the applications in a timely and coherent manner, implying a dependency on external experts. Secondly, insufficient alignment of mandates and coordination of procedures between the DoH unit responsible for market approval – a regulatory task – and the one dealing with pricing and reimbursement decisions. The latter tasks are components of typical HTA, but the general consensus was that they needed to be specified much more clearly. The combination of these two challenges has led to delays in HTA decisions and an accumulation of technologies waiting to be assessed.

To address these challenges, there was broad support from all stakeholders consulted for the further enhancement of the HTA process in Abu Dhabi. They expressed a strong sense of urgency, driven on the one hand by the recognition of high demand (partly induced) for innovative solutions in the delivery of health services and on the other hand by concerns about the sustainability of a further expansion of health services and the health benefit packages already in place. Participants in the June 2022 stakeholder workshop called for a more explicit and transparent HTA process in Abu Dhabi, along with concrete measures to address the above challenges. They recommended to obtain external expert guidance on the use of evidence-informed HTA decision making following the EDP framework and to consolidate this in a roadmap for stepwise implementation.

### Roadmap

The stakeholder consultations and the national workshop, along with d international good practices of EDP implementation as summarized by Oortwijn et al. (14), informed the development of a 5-year roadmap. It envisages the establishment and implementation of a comprehensive HTA framework in Abu Dhabi with an explicit aim, a clearly defined scope for HTA (which types of technologies qualify for assessment), appropriate governance principles, and an organizational structure for a dedicated HTA agency and committees embedded in the existing health sector set-up.

While there is no single best approach to the establishment of a national HTA agency, it was agreed that emphasis be put on general principles that support standards of transparency, good governance, and credible, evidence-informed decision-making. A key element here is the clear connection of HTA to decision making, supported by strong legal, policy, and procedural acts. It was agreed that priority be given to developing a regulatory framework that sets out governance arrangements, standard review processes based on an explicit list of HTA decision criteria, and appeal rights and procedures. As a starting point, the composition, role, and responsibilities of an independent advisory committee would be articulated, along with the status of future committee recommendations. The role and remit of a dedicated HTA agency, separate from the DoH, would be formulated, including its scope of work and the demarcation of HTA decision-making authority between the DoH and the future HTA agency.

The establishment of a temporary HTA Taskforce was proposed that would act as a steering group to define and put the most urgent steps into operation in the year to come. After careful consideration of such a taskforce, this role has since been taken up jointly by the DoH’s Research and Innovation Center and the Healthcare Payers Sector, with the objective of further developing Abu Dhabi’s HTA strategy and associated implementation framework. The initial activities include: (i) defining the decision-making framework and process for translation of HTA outcomes into policy and practice; (ii) completing the description of the organizational structure, identifying key roles and responsibilities; (iii) articulating a process map and establishing operating procedures and timelines; (iv) establishing relevant advisory committee(s), recruiting and funding as necessary; (v) allocating dedicated human and financial resources to conduct HTA; and (vi) continuing to engage relevant stakeholders, including academia, clinicians, hospital managers, industry, professional associations, and patient interest groups. If successfully undertaken by the DoH, these activities would lay the foundation for the establishment in the medium-term of a relatively small, but technically strong HTA structure, and its subsequent institutionalization.

### Proposed EDP steps

With regard to the eventual establishment of an HTA advisory committee (step A in the EDP framework), the roadmap proposed, among others, that (i) the main task of this committee was to conduct appraisals based on the evidence collected during the assessment phase using a list of decision criteria and to prepare recommendations on the public reimbursement and pricing of Thiqa-related health technologies; (ii) that the regulation (to be defined) specifies that the DoH can only make a reimbursement decision if it has obtained a recommendation from the advisory committee based on an HTA report and completed deliberations (steps D1 to D3 in the EDP framework – that is scoping, assessment, and appraisal); and (iii) that Abu Dhabi may capitalize on already existing advisory committees, such as the Health Research Technology Committee, which focuses its recommendations on safety, clinical effectiveness and ethics, and the Healthcare Payers Sector, which conducts HTAs and makes recommendations for pricing and reimbursement decisions.

As to the selection and definition of HTA decision criteria (step B in the EDP framework), the roadmap proposed, in line with the guidance provided in the EDP Guide (12), to start by reviewing the broad health system goals and values laid out in policy documents, and derive a long-list of suitable decision criteria from these; to then subject this long-list to a survey among a broad range of stakeholders; and thereafter have a smaller group of stakeholders scrutinize the long-list and turn it into a short-list.

With regard to the selection of health technologies for assessment (step C in the EDP framework), the roadmap suggested that Abu Dhabi opts for a combination of ad hoc decisions and nomination. The advice is to start small, with a specific focus, for example prioritizing HTA for reimbursement decisions (e.g., review of medicines dossiers submitted by the industry that have a high budget impact), and gradually moving toward other health technology topics selected through an explicit and transparent multistakeholder process. This may include an agenda for possible disinvestment decisions, informed by burden of disease data, budget impact estimates, and/or international practice guidelines (e.g., interventions that may be considered obsolete). This could produce some “quick wins” and at the same time enhance the legitimacy of HTA.

The roadmap further foresees formulation of an HTA program, especially the development of methodological HTA guidelines for the conduct of HTA (steps D1 and D2 in the EDP framework) and appointment of specialist staff to maintain local and national registries.

HTA capacity will need to be built through education, training, and networking, while making use of regional and international collaboration. Continuous awareness raising of the added value of HTA among its users is recommended, through periodic stakeholder consultations, circulars, and press coverage. The roadmap for HTA was discussed and agreed upon by the Department of Health in Abu Dhabi in October 2022, and a start has been made with its implementation.

## Discussion

The appetite and demand for HTA in the UAE, specifically in Abu Dhabi, is high among all stakeholders, and there is political will within the DoH of Abu Dhabi to strengthen HTA structures and procedures. This is fully in line with the growing international consensus that HTA can be instrumental in the broad process of setting priorities and allocating resources in an informed manner (29). In Abu Dhabi, HTA could help the DoH to define health benefit packages that are best suited for the population and its health system. With the goal of achieving UHC, reimbursement decisions on health technologies that offer value for money are critical for improving equitable access to healthcare, adequate financial risk protection, and ultimately good health outcomes. Institutionalizing HTA will help create an effective, more efficient, equitable, sustainable, and resilient health system, leading to the progressive realization of UHC in Abu Dhabi.

Much attention is being given to embedding HTA in the local policy and institutional context, taking on board the perspectives and interests of local stakeholders, and being cognizant of enablers and barriers associated with strengthening the HTA work that is already being done in Abu Dhabi. The experience of other countries that are in a nascent phase of HTA institutionalization may serve as examples (2–6, 30–32). In addition to what Ahmad et al reported (10), this paper introduces and explores the potential to strengthen the use of evidence-informed deliberation, following principles of transparency of HTA decisions and decision-making processes, and emphasizing the articulation of clear mandates for the organizations involved in the HTA process. Opportunities have been identified to exchange approaches towards institutionalizing HTA with policy makers and practitioners in other countries and learn from their experiences through international networks such as HTAi.

As to the involvement of stakeholders in institutionalizing HTA, it has proven challenging to secure an adequate representation of both patients and citizens (taxpayers) in the discussions that have taken place so far. This is an aspect that merits further attention, not least because from a legitimacy point of view one would expect the Government of Abu Dhabi to make an effort to secure public support for any tough decisions that it may have to take in the future in case the demand for health technologies outstrips the available budget.

Although the current limited HTA technical capacity within UAE/Abu Dhabi is broadly acknowledged, strengthening and expanding it to address the increasing demand for HTA forms another challenge. It requires a strategy and timely investments in education and training. Two recent publications about HTA in India also call for strong local ownership and contextualizing HTA development (33;34). They reflect on three strategic requirements for achieving HTA policy objectives, that is, smart growth, technical excellence, and transformative influence. These considerations appear relevant for any context where HTA seeks a suitable path to maturity and impact, including Abu Dhabi.

The main strength of this work is that it demonstrates and reconfirms the importance of political will and the leveraging role of academia and of international collaboration among – what Ahmad and colleagues called – “HTA policy entrepreneurs” (10) in different phases of HTA implementation, the maturing of HTA processes, and the institutionalization of HTA structures. The fact that the stakeholder representatives that were interviewed and took part in the workshop in early June 2022 had all been invited by the DoH forms a limitation. It may have created a selection bias. A Hawthorne effect may also not be ruled out, as all face-to-face interviews were held at the DoH premises, with one or two DoH staff attending several of the group interviews as observers. Two DoH staff members also attended the online interview session with industry representatives, as observers.

## Conclusion

Broad HTA stakeholder consultation has been instrumental toward the establishment of a comprehensive HTA framework in Abu Dhabi and the development of a road map. With time, it will become clear to what the extent the proposed EDP steps will actually be implemented and help address the challenges identified in the HTA set-up and the processes already in place. The eventual HTA framework needs to have an explicit aim, a clearly defined scope for HTA (indicating which types of technologies will be assessed), appropriate governance principles, and an organizational structure dedicated to HTA with committees embedded in the existing health sector set-up.

Technical work regarding the conduct of assessments needs to be complemented by advocacy, consultation, and the involvement of relevant stakeholders throughout the EDP process. The interest raised during the stakeholder consultations and the commitments made hold promise for the firm adoption and establishment of EDP principles to support HTA in Abu Dhabi that have potential to contribute to a sustainable high-quality healthcare system. A momentum for HTA institutionalization has been created which needs to be sustained through a gradual implementation of the road map, accompanied by continued stakeholder participation.

## Supporting information

Bijlmakers et al. supplementary material Bijlmakers et al. supplementary material

## Supplementary material

The supplementary material for this article can be found at http://doi.org/10.1017/S0266462324004744.
