# Supplementary material for: Road toward institutionalizing health technology assessment in the Emirate of Abu Dhabi: The role of evidence-informed deliberative processes
Source: Int J Technol Assess Health Care. 2024 Dec 18;40(1):e80. doi: 10.1017/S0266462324004744 (PMC11703617; doi:10.1017/S0266462324004744)
Supplement: Bijlmakers et al. supplementary material [file S0266462324004744sup001.docx]

**Supplementary material: Topic list for semi-structured interviews**

**P1 – Interview schedule for meeting with Policy makers**

1. *a. What have been the main HTA-related achievements (highlights, advancements, results) over the past 2-3 years in UAE? Please give examples.*

*b. What have been the main constraints (limitations)?*

1. *a. Are you familiar with HTA processes and results in other countries?*

*If yes, have you ever used (or referred to) an HTA report produced by another country/agency?*

*b. Do you know of countries that have HTA models in place that UAE could emulate or learn from?*

*Which ones and why ?*

1. *For what purpose would you like to use HTA in UAE?*

e.g. Conduct assessments of individual interventions; Enable proper pricing & reimbursement decisions; Improve procurement procedures; Whole benefit package revisions, Partial benefit package revisions (e.g. per condition or clusters of interventions).

1. *a. What would you envisage for HTA development in UAE in the short term, say the next 2-3 years?*

*b. What would be the preferred status of HTA in UAE in 5 or 10 years’ time?*

1. *What do you think is the biggest threat if HTA was not further developed in the years to come?*

Our understanding of the current situation at DoH is as follows:

*Health Technology & Innovation Impact Assessment Section (part of Research and Innovation Center – section head: Amna Al Saeedi) provides (for 2 year) technical evaluation of new technologies, mainly looking at safety and risk-benefit, giving market access approval to the entire market (all patients, public and private providers). **This is a regulatory task (but in DOH it is referred to as HTA).**

1. *Do you consider/envisage the establishment of a new dedicated HTA entity/unit?*

#### *a. What should its role/mandate be?*

*b. How would this role/mandate relate to the existing Govt/DOH processes?*

*c. Where should it be placed in the Govt’s/DOH’s organigram (or: to whom should it report)?*

*d. What are the (potential) synergies between this HTA entity/unit and the existing DOH structures?*

*e. What are the (potential) synergies with other actors/stakeholders?*

##### Would there be need for some kind of legal framework to link HTA to decision making?

1. *Q’s on the six EDP steps towards an HTA process that would result in legitimate decision making: (a) current situation versus (b) ideal situation; and (c) the possible need for guidance.*
   1. *Installation of an advisory committee*
   2. *Definition of decision criteria*
   3. *Selection of health technologies for HTA*
   4. *A Scoping B Assessment C Appraisal*
   5. *Communication and appeal*
   6. *M&E*

##### What are your expectations for the workshop on Friday and thereafter?

##### (think for example of technical guidance, training, …)

**P2 – Interview schedule for meeting with Payers (Daman only)**

1. *a. Are you familiar with HTA processes and results in other countries?*

*If yes, have you ever used (or referred to) an HTA report produced by another country/agency?*

*b. Do you know of countries that have HTA models in place that UAE could emulate or learn from?*

*Which ones and why ?*

1. *a. From your agency’s/organisation’s/institution’s perspective have there been any major HTA achievements (highlights, advancements) in the past 2-3 years in UAE? If yes, please give examples.*

*b. What have been the main constraints (limitations)?*

1. *From your agency’s perspective, would you like to see HTA being further developed in UAE?*

*Which aspect of HTA in particular? For what purpose?*

1. *a. What would you envisage for HTA development in UAE in the short term, say the next 2-3 years?*

*b. What would be the preferred status of HTA in UAE in 5 or 10 years’ time?*

1. *What do you think is the biggest threat if HTA was not further developed in the years to come?*
2. *Would your agency endorse the establishment of a new, dedicated HTA entity/unit?*

*a. If yes, why?*

*b. Should this unit be placed within the DoH or outside? Why?*

#### *b. What should its role/mandate be?*

*c. What advantage or disadvantage) would it have for your agency/organisation?*

1. *Q’s on the six EDP steps towards an HTA process that would result in legitimate decision making: (a) current situation versus (b) ideal situation; and (c) the possible need for guidance.*
   1. *Installation of an advisory committee*
   2. *Definition of decision criteria*
   3. *Selection of health technologies for HTA*
   4. *A Scoping B Assessment C Appraisal*
   5. *Communication and appeal*
   6. *M&E*

##### What are your expectations for the workshop on Friday and thereafter?

##### Do you see any particular role for the agency that you work for in the (further) development of HTA in the years to come?

**P3 – Interview schedule for meeting with Product makers (manufacturers)**

##### As in P2

**P4 – Interview schedule for meeting with Principal investigators**

##### Start by finding out in what way the PIs have been involved in HTA in UAE: their role in teaching, research, consultancy.

##### Then questions as in P2.

**P5 – Interview schedule for meeting with Patient representatives (and Public)**

##### Start by finding out what the interviewees’ role is in serving/defending patient interests.

##### How do they work?

##### Ask for examples of recent successes (or failures).

##### Strengths, weaknesses, opportunities, threats – if any.

##### Then questions as in P2

**P6 – Interview schedule for meeting with Providers / health professionals**

##### As in P2

**P7 – Interview schedule for meeting with Purchasers**

##### As in P2
